# Enhanced Breathing Pattern Detection during Running Using Wearable Sensors

**DOI:** 10.3390/s21165606

**Published:** 2021-08-20

**Authors:** Eric Harbour, Michael Lasshofer, Matteo Genitrini, Hermann Schwameder

**Affiliations:** Department of Sport and Exercise Science, University of Salzburg, Schlossallee 49, 5400 Hallein-Rif, Austria; michael.lasshofer@plus.ac.at (M.L.); matteo.genitrini@plus.ac.at (M.G.); hermann.schwameder@plus.ac.at (H.S.)

**Keywords:** breathing pattern, breathing rate, respiratory frequency, ventilation, breathing sensors, respiration sensors, running sensors, respiratory inductance plethysmography

## Abstract

Breathing pattern (BP) is related to key psychophysiological and performance variables during exercise. Modern wearable sensors and data analysis techniques facilitate BP analysis during running but are lacking crucial validation steps in their deployment. Thus, we sought to evaluate a wearable garment with respiratory inductance plethysmography (RIP) sensors in combination with a custom-built algorithm versus a reference spirometry system to determine its concurrent validity in detecting flow reversals (FR) and BP. Twelve runners completed an incremental running protocol to exhaustion with synchronized spirometry and RIP sensors. An algorithm was developed to filter, segment, and enrich the RIP data for FR and BP estimation. The algorithm successfully identified over 99% of FR with an average time lag of 0.018 s (−0.067,0.104) after the reference system. Breathing rate (BR) estimation had low mean absolute percent error (MAPE = 2.74 [0.00,5.99]), but other BP components had variable accuracy. The proposed system is valid and practically useful for applications of BP assessment in the field, especially when measuring abrupt changes in BR. More studies are needed to improve BP timing estimation and utilize abdominal RIP during running.

## 1. Introduction

### 1.1. The Importance of Breathing Pattern

Breathing pattern (BP) is a comprehensive conceptual framework that provides valuable insights for broad applications, such as sports performance and clinical medicine. Breathing rate (BR), also known as respiratory frequency, is one simple, popular component of BP that has earned special attention recently for its relevance as a psychophysiological biomarker [1]. BR is strongly correlated to physical workload, is easy to measure, and is particularly information-rich in exercise settings [2]. Other components of BP, such as timing, drive, and coordination, contain additional information relevant to exercise, and their measurement may help to improve performance or identify BP disorders [3,4]. One additional component of BP of particular relevance to rhythmic exercise (e.g., running) is locomotor-respiratory coupling (LRC). LRC is an entrainment phenomenon common in endurance running and other activities, and it may reflect optimal states in the self-organizing complex system of breathing regulation [5]. However, LRC, as well as many other components of BP, are hard to measure in real-world conditions without specialized equipment and expert knowledge.

### 1.2. Breathing Sensors

Recent progress in wearable sensors has expanded the capabilities of BP analysis during exercise. BP data can be extracted from many sensor types, such as stretch sensors, electrocardiogram, and microphones. The former appear to be most promising to measure BP during activities with motion and noise artifact, such as running [6]. Indeed, several studies have demonstrated that stretch sensors such as inductive and capacitive sensors are robust to motion artifact, unobtrusive, and can accurately measure BR, thoracolumbar asynchrony (a measure of coordination), and minute ventilation [6,7]. Not only are they valuable for data collection quality, but also expand the possibilities of field analysis in natural running settings by avoiding the expense and obtrusiveness of traditional spirometry breath analysis.

The Hexoskin garments (Carre Technologies, Canada) are wearable shirts with embedded sensors for monitoring vital signs including BP. The Hexoskin smart shirt (HX) was developed for use in elite sport and clinical settings and has sensors for activity (3-axis accelerometer, ±16 g, 64 Hz), respiration (dual 2-channel respiratory inductance plethysmography (RIP), 16 bit, 128 Hz), and heart sensors (1-lead ECG, 12 bit, 256 Hz). The company states that the respiration sensors are accurate between 3–80 bpm, 80 mL–10 L, and 2–150 L/min, and with 8 ms resolution. Of the 42 publications on HX published at time of writing, only a handful evaluated the HX for respiration detection accuracy, mostly concluding that it is a valid measurement device for BR [8,9,10].

### 1.3. Flow Reversal Detection

One advantage of RIP sensors, such as those in HX, is the possibility of precise flow reversal (FR, also known as onset of inspiration or expiration). This information can be used to calculate not only the exact breath cycle time (tB) and instantaneous BR, but also metrics such as timing (e.g., inhale time, duty cycle) and LRC (when combined with step information). Since HX also includes an accelerometer, it is well-suited for LRC detection.

Previous validation studies of the HX have yet to examine this sensor for FR detection. Although FR timestamps are provided in the company’s exported metrics, the event detection methods are unpublished. A suitable signal processing algorithm for FR detection is, therefore, warranted to examine the HX for this capability and to enable future use cases such as LRC analysis. A recent comprehensive systematic review identified several viable FR and BP detection algorithms based on commercial and custom stretch sensors [11]. The authors concluded that peak analysis is a robust, lightweight, and accurate method of FR detection ideal for these sensor types.

To evaluate the HX and a custom algorithm for these purposes, it is essential that its accuracy is evaluated versus a reference device. Such studies are generally performed in a laboratory setting using a spirometer as the reference gold-standard [6]. The turbine flowmeter inside a spirometer can be used to determine the exact time of FR and is robust to artifact. Accurate FR detection would enable the HX to measure LRC when combined with its accelerometer data, but the accuracy of its detection greatly affects LRC calculation. For example, during intense running when BR could approach 50 bpm, an FR detection delay of 0.2 s could misrepresent LRC phase synchronization by 15%. Therefore, it is essential to calculate the FR detection accuracy before using the HX, or any similar system, for LRC calculation. Furthermore, quality FR detection can be used for BP analysis, which also requires validity assessment before further use.

Hence, the purpose of this study was twofold:

1. To determine the accuracy and precision of the HX and custom algorithm for detecting FR during running;
2. To determine the measurement agreement of the HX and reference spirometer for measuring BR and timing during running.

## 2. Materials and Methods

The University Ethics board approved this study and informed consent was obtained from all participants. This investigation consisted of a single incremental treadmill test until volitional exhaustion in a sports physiology laboratory. Data were concurrently collected from multiple synchronized sensor systems.

### 2.1. Participants

A convenience sample of 12 participants (6 m/6 f) was recruited for this study (Table 1). They had diverse sport experience with at least 2 years running experience. Participants were asked about any breathing irregularities or techniques employed during exercise. Anthropometric data, including torso circumference and torso skinfolds (supraspinale, iliocristale, abdominale), were collected by a certified ISAK-2 practitioner.

### 2.2. Instruments

**Figure 1:**
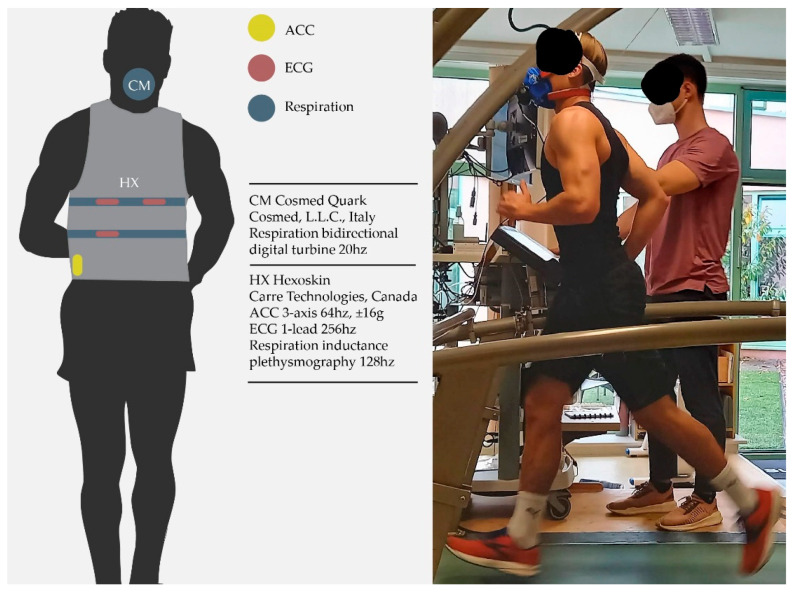
Experimental sensor setup with reference spirometer setup and Hexoskin smart shirt. ACC: accelerometer, ECG: electrocardiogram, CM: Cosmed, HX: Hexoskin.

Participants were fitted with a Hexoskin smart shirt according to the manufacturer’s recommendations (Figure 1). The manufacturer’s provided elastic support bands were placed over the thoracic and abdominal respiration sensors to minimize movement artifact during running. Raw respiration data were exported from the Hexoskin Hxservices software for post-processing.

During the treadmill test, the Cosmed Quark spiroergometry system (CM; Cosmed, Rome, Italy) was used as the reference system for respiration data. Specifically, flow data from the mouth turbine (bidirectional digital turbine, 28 mm, 0.03–20 L/s) were collected at 20 Hz to obtain the raw signal for processing. The system was calibrated according to the manufacturer’s recommendations and has a claimed accuracy of ±2%. Each mask was fitted to the participant by a trained exercise physiologist. Factory processed data were exported in breath-by-breath mode (henceforth referred to as “factory” data) from the CM Omnia software for later analysis.

### 2.3. Data Collection

After an introduction and sensor fitting, participants completed a 10 min warmup walking and jogging (4 km/h and 7 km/h, respectively) on a treadmill (h/p cosmos sports, Traunstein, Germany). The treadmill was set to 1% gradient for the duration of the test.

**Figure 2:**
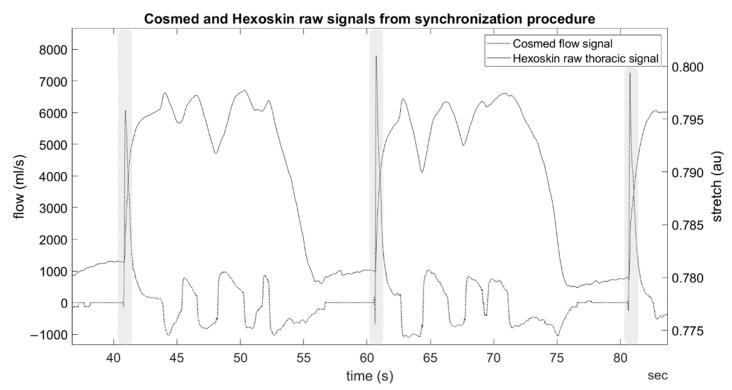
Example synchronization procedure sensor signals from one participant. Shaded areas indicate location of forced exhale within procedure. Au: arbitrary units.

To synchronize the respiration systems, a cycle of three apneas and forced exhalations was performed. Each participant was familiarized with the procedure in order to perform each maximal exhalation consistently. This routine produced a characteristic signal (Figure 2) that was later used to synchronize the systems to within a time frame of <0.05 s. The synchronization was performed before and after the experiment to confirm uniform sampling and lack of drift.

After synchronization, the incremental treadmill test began at a speed of 8 km/h. The speed was increased by 1 km/h each minute until volitional exhaustion. This test was selected to expose each participant to a range of intensities and, thus, breathing patterns from low intensity until maximal exertion.

### 2.4. Data Processing

Raw CM flow and HX thoracic signal data were exported in .csv format and processed via a custom MATLAB routine (MATLAB R2021a, The MathWorks Inc., Natick, Massachusetts, USA). First, the signals were upsampled via linear interpolation to a common frequency (128 Hz). Then, synchronization was performed by detecting the average time lag between all three peaks in the CM flow signal versus the peaks in the first derivative of the HX thoracic signal (Figure 2). The mean time lag was used to calculate the synchronization value, and the standard deviation of the time lags was used to calculate a synchronization error value. The difference between the first and second sync was calculated to quantify any sensor drift, and any detected drift was corrected manually.

The data were trimmed from the start to the end of the running trial. The entire raw waveform from the start to end of the test was retained, as the FR detection was not directly affected by changes in treadmill speed.

**Figure 3:**
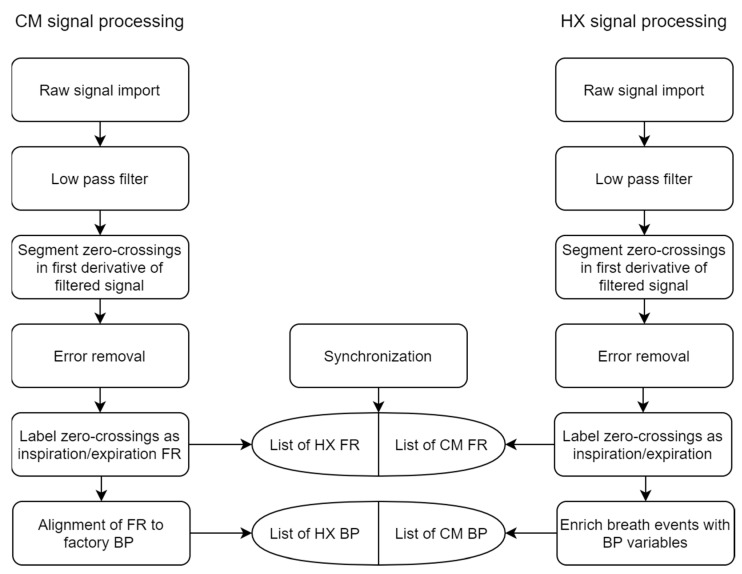
Flowchart of signal and data processing. FR: flow reversal, BP: breathing pattern.

**Figure 4:**
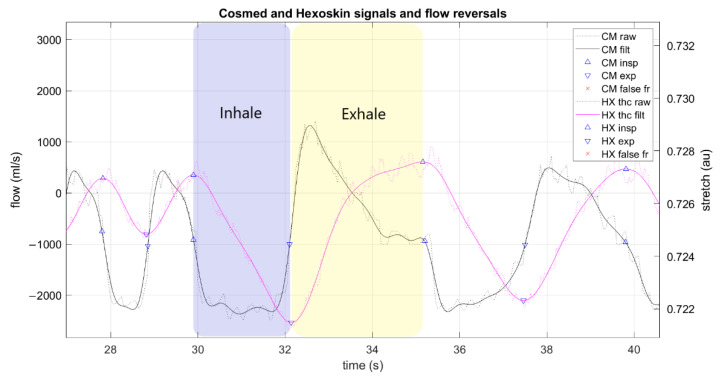
Example Cosmed and Hexoskin signals and detected flow reversals from one trial. No false flow reversals were detected in this signal example. Annotated “exhale” and “inhale” breath phases are approximate representations of breath phase determination by the algorithm. CM: Cosmed, HX: Hexoskin, filt: filtered, insp: inspiration, exp: expiration, au: arbitrary units; fr: flow reversal.

**Figure 5:**
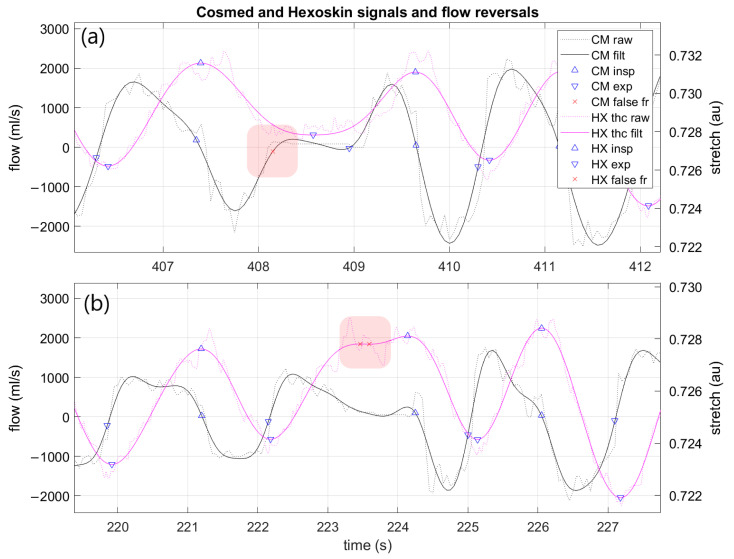
Example Cosmed and Hexoskin signals and detected flow reversals from one trial. Note false flow reversals labeled for removal. Cosmed error is labeled in (**a**) and Hexoskin false positives in (**b**). CM: Cosmed, HX: Hexoskin, filt: filtered, insp: inspiration, exp: expiration, fr: flow reversal.

The raw CM flow data were processed to calculate ground truth FR timestamps per breath. This was necessary because the factory data format label each breath to the nearest whole second; a custom approach was used to leverage the maximum available sample rate in the flowmeter (20 Hz). Thus, the raw data were processed via the following steps: filter, segment, error correction, event labeling, and alignment with factory-processed data (Figure 3). Raw data were filtered with a 4th order Butterworth low-pass filter and MATLAB function “filtfilt” to prevent any phase distortion. The filter cutoff frequency was automatically detected via Hamming window to capture 95% of the signal power spectrum (reported in results). Then, zero crossings were detected from the filtered signal corresponding to areas of “zero flow” (0 mL/s) at each FR (Figure 4). Then, possible false events detected were flagged according to several heuristics. If a zero crossing was detected 1) where the raw, unfiltered signal did not actually cross zero or 2) the signal had a long duration (≥0.25 s) at 0 mL/s, then the event was marked as possible false for later classification (Figure 5). The “false” events were then manually checked by an expert before elimination to verify whether the event was a “true” breath or indeed “false” and confirmed by the factory data. The list of FR was labeled as inspiration or expiration based on the signal slope being negative or positive at each event, respectively. Finally, each FR was automatically aligned to the corresponding breath in the CM factory data by examining the correlation between three features: event timestamp, tB, and ds (duty cycle). Data were manually checked for any discrepancies. This produced a comprehensive dataset with precise FR timestamps and matched CM factory data features (tB, ds).

Raw HX data were analyzed in a similar routine as above. Signals were filtered with the same procedure as CM; then, zero crossings were detected in the first derivative of the thoracic signal, as each FR occurs near signal peaks and valleys (Figure 4). For error correction, a routine similar to that reported by [12] was implemented; each FR was “scored” via three characteristics: (1) time from last FR, (2) positive or negative slope, and (3) signal magnitude. Then, FR were flagged as “false” if: (1) more than 60% shorter than the average time difference of last five FR or (2) they were first in a short (<0.5 s) double event (same slope sign) (Figure 5). These “false” events were manually checked before elimination. Each FR was labeled as inspiration or expiration according to the signal peaks and valleys, respectively. The list of FR was then used to enrich the dataset with the following features for each breath: time of inhale (tI; time from each peak to valley), time of exhale (tE; time from each valley to peak), tB (sum tI+tE), BR (60/tB), and ds (tI/tB). BR variability (BRV) was calculated using the coefficient of variation (CV) of tB over five breaths [13,14].

### 2.5. Statistical Analysis

The HX and CM were compared for FR detection and BP detection.

#### 2.5.1. FR Detection

In order to validate the HX and proposed algorithm for FR event detection, three criteria were considered, as in [15]: ratio, precision, and recall.

Ratio: the quotient between the number of FR detected by the HX algorithm and the number of FR labeled in the CM. If the ratio is >1, the breath detection algorithm overestimates the number of actual FR, and vice versa. A ratio of one means that the number of detected FR and actual FR are the same, indicating a good algorithm performance. However, it does not indicate if all the detected FR were actual FR, since only the number of FR is evaluated.

- Precision: the proportion of FR detected by the algorithm that are actual FR (Equation (1)).
- Recall: the proportion of actual FR detected by the breath detection algorithm (Equation (2)).

In all metrics, a value of 1 means perfect performance. To calculate the FR count precision and recall values, the following metrics were determined:

- True positives (TP) are determined by a detected FR that was an actual FR (based on CM factory breath-by-breath data). A correct detection is characterized by the correct breath phase determination (either inspiration or expiration) and by the difference in time between the detected and the actual timestamp. If the time difference is smaller than half the mean tB of the last five breaths, it is a true positive.
- False positives (FP) reflect the number of FR detected by the breath detection algorithm that are not actual FR detected by the reference system.
- False negatives (FN) are the number of actual FR that are not detected by the breath detection algorithm.

Precision = TP × (TP + FP)^−1^ (1)

Recall = TP × (TP + FN)^−1^ (2)

From the list of matched true positive FR, the absolute timestamp of each system was compared for equivalence. The time difference for each FR was calculated to determine the precision of HX FR detection, where a negative value indicates that the HX detected the FR before the CM, and vice versa. Because the CM collects at a maximum sample frequency of 20 Hz, the maximum precision that could be estimated is ±0.05 s in perfect equivalence. Mean relative and absolute percent error (MRPE and MAPE, respectively) were calculated to quantify the magnitude of measurement error across different tB values. Levene’s test was used to detect significant differences in lag distribution differences between breath phase (inspiration and expiration) and participants. The a priori acceptable error for FR was set to 10%, where <10% MAPE was considered “acceptable” and >10% “unacceptable” [16].

#### 2.5.2. BP Detection

We followed the recommendations of [11] to compare BP detection between the HX and CM using absolute and relative errors, Bland–Altman analysis with limits of agreement (LOA) [17], correlation coefficients, and regression. The combined list of all breaths was used to compare the calculated BP variables tI, tE, tB, and ds between devices. BR was low-pass filtered at 0.04 Hz before method comparison as recommended by [18].

In all comparisons, the CM factory data and the HX data from the custom algorithm was used. Each breath was considered an independent event, and data were pooled for comparison.

Statistical analysis was performed using XLSTAT 2021.2.2 (Addinsoft, New York, NY, USA). All breaths were compared for agreement via Pearson’s correlation, Passing–Bablok regression [19], Bland–Altman plots, and paired t-tests. Correlation coefficients were interpreted as follows: 0.00–0.10 negligible, 0.10–0.39 weak, 0.40–0.69 moderate, 0.70–0.89 strong, and 0.90–1.00 very strong [20]. Shapiro–Wilk tests were used to confirm normal distributions, and Spearman correlations, Wilcoxon signed rank, and Levene’s tests were used in case of a non-normal distributions. MRPE and MAPE were calculated to quantify the magnitude of measurement error relative to the criterion. Significance was set to α = 0.05 unless otherwise stated. All data are listed as mean and 95% confidence interval unless otherwise stated.

## 3. Results

### 3.1. Sample

The participants ran for an average 523 ± 99 s (Mean ± SD), recording 325 ± 78 breaths. Group BR ranged from 28.4 ± 7.0 breaths per minute (bpm) at test start up to 53.1±6.4 bpm at test end. This produced a dataset of 7816 FR and 3907 breath events for analysis (Table 2). The filter was adjusted to 0.84 Hz for this sample.

### 3.2. FR Detection

Out of 7816 FR identified by the CM, the HX algorithm correctly identified 7805 (Table 3). The HX also detected 15 false events that were not recorded by the CM. Thus, the precision was 0.998, the recall was 0.998, and the ratio was also 0.998.

**Figure 6:**
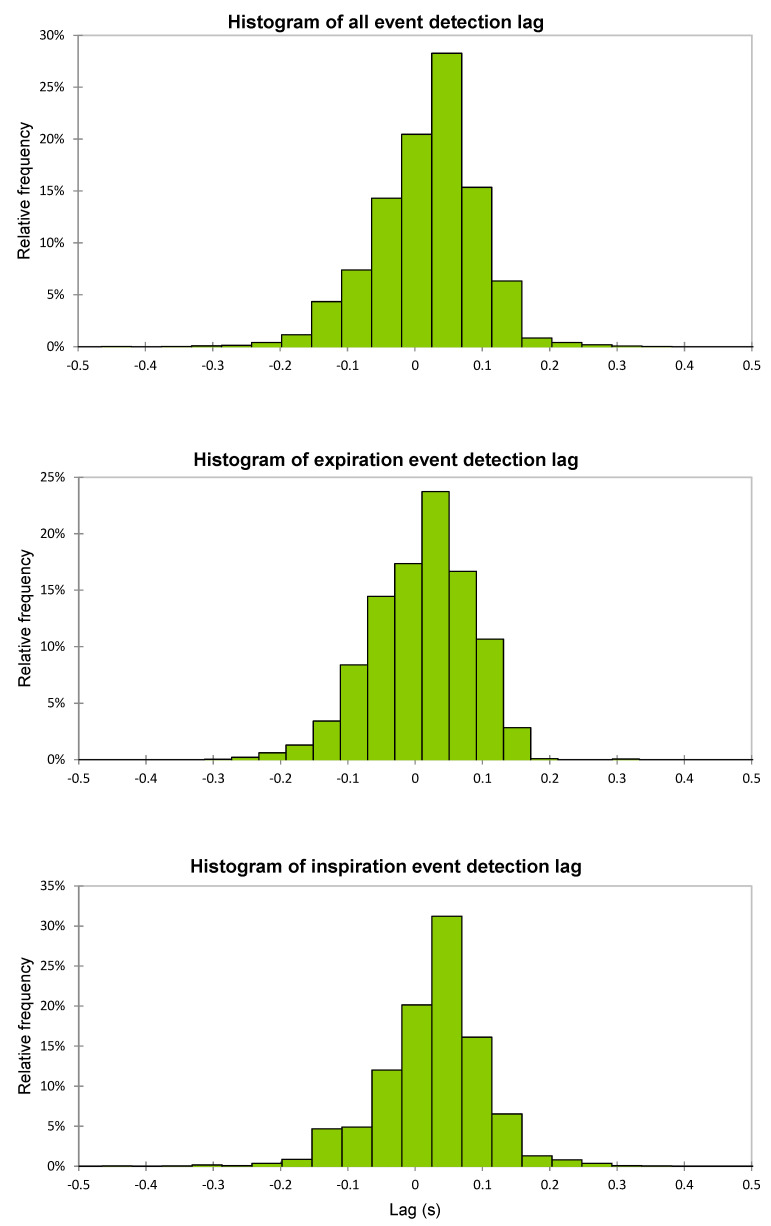
Histogram of event detection lags for full dataset, expiration, and inspiration events.

Upon closer inspection, the HX algorithm detected each FR within <0.1 s compared to the CM (lag = 0.018 s [−0.067,0.104]) (Table 3). The distribution was non-normal (W = 0.93, *p* < 0.001) and skewed negative (Figure 6); Levene’s test revealed significant inter-individual variation in lag between individuals (F(9,7814) = 17.69, *p* < 0.001). Another Levene’s test determined that there was no significant difference between the lag differences on inspiration FR versus expiration FR (F(1,7814) = 0.59, *p* = 0.45). There was a weak negative relationship between the FR detection lag and tB (r = −0.11, *p* < 0.001), suggesting that the lag is almost constant across various BR. When adjusted to tB, the MRPE was 1.19% (−3.83,6.20) and the MAPE was 4.05% (0.86,7.24).

### 3.3. BP Detection

**Figure 7:**
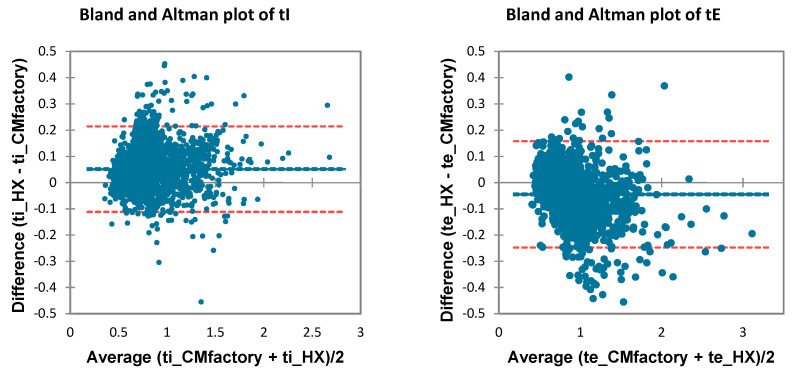

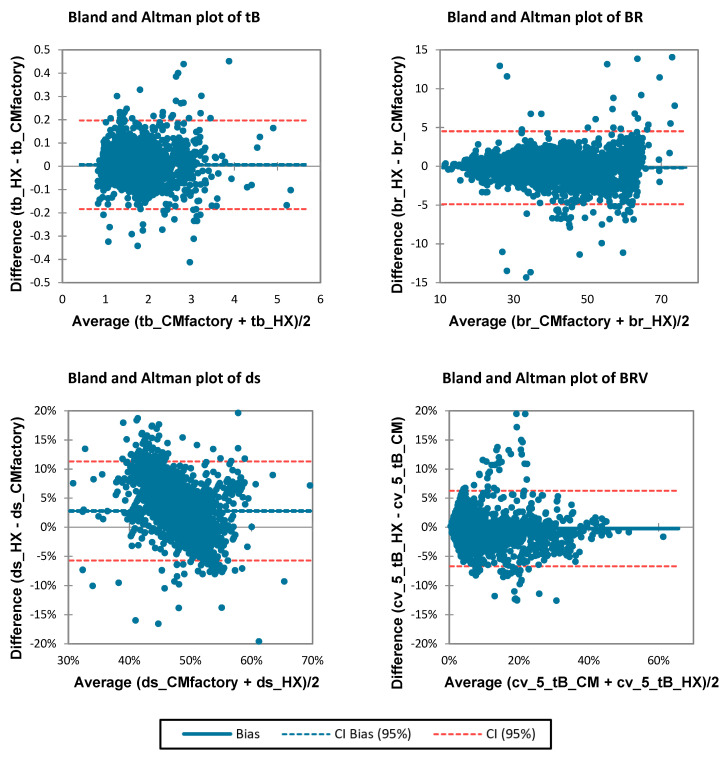
Bland–Altman plots of all BP comparisons. CI: 95% confidence interval.

**Figure 8:**
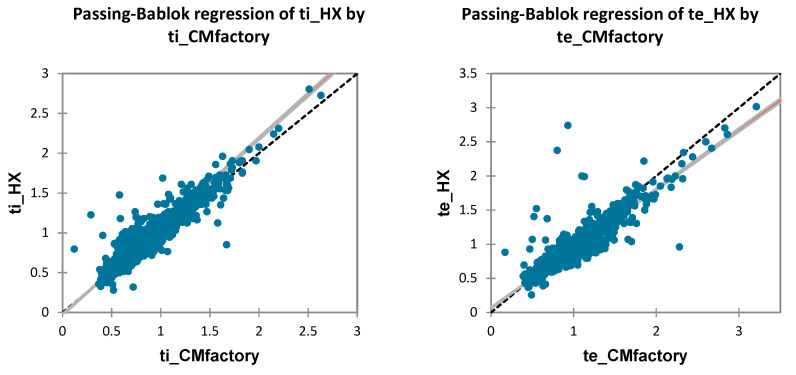

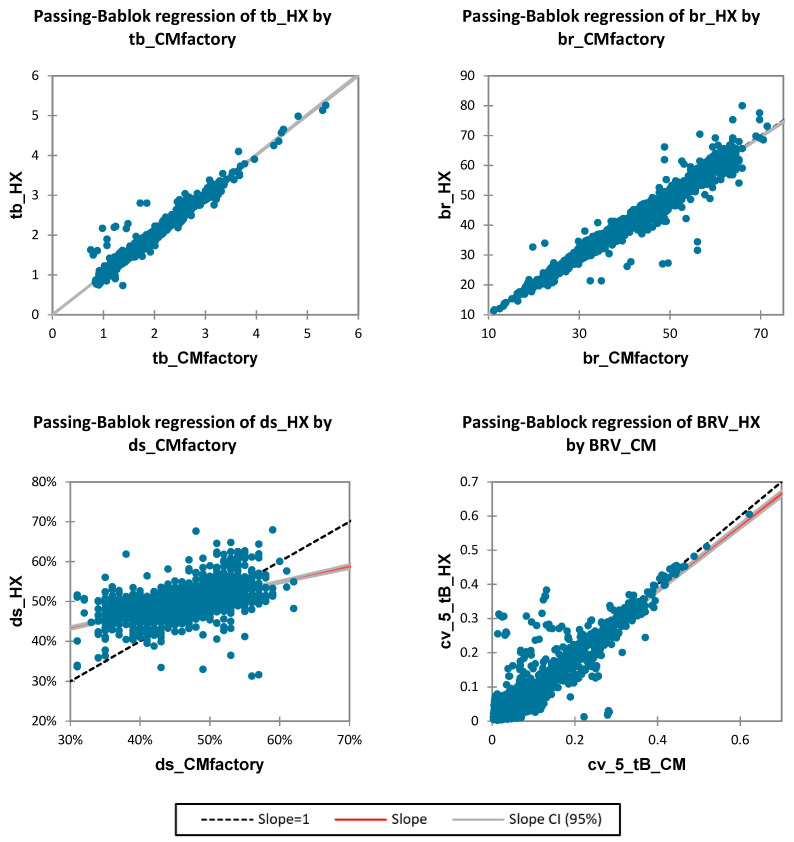
Passing–Bablok regression plots of all BP comparisons. CI: 95% confidence interval.

CM and HX BP detection was largely in agreement, with correlations >0.9 and MAPE <10% for four out of six comparisons (Table 4). tB and BR detection were very accurate, with low bias (0.007s and −0.19 bpm, respectively), MAPE (2.74%), and acceptable LOA less than ±5% (Figure 7). Notably, ds detection was not in agreement; despite bias and error, Passing–Bablock regression revealed systematic error (slope = 0.39 [0.33,0.45]) for the HX across increasing ds (Figure 8). BRV agreement between devices had mixed results: absolute bias and LOA were low (−0.2 [−6.72,6.26]) but relative differences were high, especially at lower values of BRV (MAPE = 36.24% [0.91,71.57]).

## 4. Discussion

The purpose of this study was to evaluate the HX and custom algorithm for FR and BP detection versus a reference system during running. Although previous studies have demonstrated the efficacy of many systems for this purpose, few have examined FR detection specifically. Although BR estimation can be reliably performed with other methods such as frequency-domain analysis, this event-based approach allows for enhanced BP analysis including breath ratio, entrainment phenomena (such as LRC), and an estimation of BRV (such as in periodic breathing) [21]. Our results suggest that the HX and custom algorithm can be successfully leveraged to determine most of these metrics during running.

### 4.1. FR Detection Accuracy

The HX and custom algorithm correctly identified over 99% of all breath events in the dataset. This verification is essential, as false detections could substantially affect subsequent calculations. Moreover, there appears to be little or no effect of FR phase (inspiration or expiration), BR, or running intensity upon event detection. It is possible that signal processing event detection techniques are negatively affected by such factors, as they can alter various signal characteristics; however, this did not occur in this study.

Stretch sensors are vulnerable to motion artifact, especially during running. The HX raw thoracic sensor signal has an acceptable signal-to-noise ratio made better with digital filtering (15.0 ± 5.2 to 34.2 ± 7.7 dB on average, respectively). This was not the case with the abdominal signal. This is expected, as the thoracic sensor is located over the xiphoid process and other tissues with low adiposity. In contrast, the abdominal sensor is located over the abdominal viscera, where higher levels of subcutaneous body fat act contribute to the “visceral piston” and introduce substantial signal noise at the step frequency [5]. The signal-to-noise ratio of the abdominal signal was negative for all participants in this study and moderately correlated to the sum of torso skinfolds (r = 0.66, *p* < 0.05). Therefore, we decided not to utilize the abdominal respiration signal for our analysis. This has practical implications for utilizing the abdominal sensor for FR detection or thoraco-lumbar coordination estimation. Although other studies have utilized similar systems for this analysis [22], we would suggest carefully considering and reporting this signal noise as part of such studies.

The automated error correction step in the proposed algorithm addressed other unique event detection problems. The simple zero-crossing breath detection step is overly sensitive, flagging many false positives despite operating on the previously filtered signal. However, the error correction removed an average of 5.7 ± 4.2 false positives for each subject, successfully increasing the precision of the breath detection. We observed that these false positives often occurred around segments of signal plateaus, which previous evidence suggests could be caused by coughing or swallowing [2]. Therefore, we designed a processing step to remove any “false” breaths detected during such events, and to only retain the last event detected in these short series. We did not observe that any “true” events were removed by this error processing step.

Our results indicate that the event detection time lag between methods was within acceptable limits (MAPE = 4.1% [0.86,7.24]). Higher error values near 10% can drastically affect subsequent calculations of BP, so acceptable error should be considered context-specific with respect to clinically relevant criteria [23]. Because the CM actually had a lower sample rate than the HX, these reported differences should be considered approximate. Rather, the CM sample rate of 20 Hz implies that each breath can be detected within ±0.025 s of the actual FR moment. At a BR = 35, this would equal about 1.55% MRPE in FR detection. Therefore, there may be additional random error contained in our estimated measurement error, and this should be considered in future applications. Other method comparisons for FR detection could utilize spirometry systems with higher sample rates.

### 4.2. BP Detection Accuracy

When compared with previous HX validity studies, our results show equal or improved accuracy of the HX and custom algorithm to measure BR. These data had an average r = 0.98 and MAPE = 2.9 ± 4.7% between the HX and CM, which is similar to correlations reported under similar conditions by [8] (range r = 0.91–0.99) and [9] (r = 0.96–0.99), higher than that reported by [10] (r = 0.68–0.94), and much lower error than that reported by Montoye, Mitrzyk, and Molesky [16] (mean ± sd = 17.4 ± 4.1%). Studies of HX validity for BR during walking [24] and cycling [25,26,27] reported similarly high concurrent validity, suggesting that the HX and custom algorithm is quite accurate for estimating BR across a variety of intensities and activities.

Recent findings suggest that BR is a critical indicator in sports performance and health, as it responds rapidly and is sensitive to changes in workload, effort, and cognitive fatigue [1,28]. Thus, a system to detect rapid changes in BR could augment future investigations. Previously proposed window-based approaches might miss rapid changes in BR and fail to characterize its true variability and complexity [29]. Breath-by-breath analysis systems such as proposed here could be used to accurately capture the short-term complexity of respiration during intense sports or other domains.

We chose to analyze and report the agreement between CM and HX on a per-breath basis despite methodological issues of dependent samples and error accumulation. More specifically, sequential event detection lag can cause negative tB bias at one breath and positive at the next. Therefore, our reported error values in tB and BR should be interpreted with caution. Nevertheless, we chose to perform this analysis because, as described in the previous paragraph, BR is a key variable for practitioners, and per-breath accuracy enables greater time-sensitive analysis. The event detection lag was small and centered around 0 s (−0.067,0.104), so any error accumulation might be considered negligible in this context. The other BP variables reported in these data would not be subject to the same error accumulation since they are indeed independent events (not sequential FR).

Despite the potential for the HX and algorithm to calculate the BP components of timing (tI, tE, ds) and BRV, our results did not show good agreement with the criterion. This is perhaps caused by HX overestimating tI and underestimating tE systematically at longer tB (Figure 8). However, this error does not explain the lack of agreement in BRV, since it is calculated per-breath from the CV of the previous five tB. Individual analysis indicated that two individuals had significant tB deviations reflecting periodic deep breathing while running; this was seen to substantially increase the relative measurement error within this sample. Rather, the CM and HX differed in how they measured extra-long breaths. This behavior is not simply a statistical outlier, but indeed reflects an important clinical phenomenon that is reflected in BRV [30]. Periodic deep breaths cause measurement method differences; more sensitive techniques are needed to detect these important BP phenomena and qualify their measurement accuracy, such as machine learning or various complexity metrics [29].

The measurement error for BRV and ds might be attributed by the different actual event phenomena detected by the CM and HX. Specifically, CM measures FR directly only centimeters away from the mouth and nose; HX detects change in ribcage dimensions. Although any FR detection lag should be reflected in our calculations, it is possible that the ds could also be affected by coordination differences when the abdominal ribcage contracts or expands before or after the thoracic ribcage [7]. We chose not to report this coordination because of significant signal noise present in the abdominal signal at the step frequency. Qualitative signal observation show that this noise dramatically affects the FR detection in the abdominal sensor signal. Thus, analysis of the abdominal breathing signal could provide valuable insights to BP analysis; however, future investigations planning to do so should report the signal-to-noise ratio and interpret results regarding BP timing, coordination, and abdominal breathing during activity with caution.

The HX and other stretch sensors are capable of estimating tidal volume and minute ventilation, although with debated accuracy. Two studies report poor agreement of HX for measuring tidal volume (TV) and minute ventilation compared to reference values during running [10,16], while another reports high agreement [24] and another mixed agreement between males and females [8]. Other studies have proposed reference-free calibration models for TV [31] and minute ventilation [27]; however, we chose not to replicate their methods on the relatively small sample size present in this study, although we do recommend evaluating those methods for such purposes.

Future investigations could examine sensors and algorithms such as that proposed here to examine other BP variables. For example, ventilatory drive (TV/tI) is another important component of BP and is considered a primary response variable to exercise intensity [4]. Combining the TV calibration proposed by [31] and the accurate tI measurement reported here, this could be extracted for other purposes in subsequent studies.

As described above, two or more respiration sensors, such as those in HX and other commercial or custom wearables, could be used to derive coordination elements of BP. One is thoracolumbar coordination; this has been measured before [7,22] but requires more evaluation during running. Another variable of interest to researchers may be “coordinative depth”, calculated via the ratio of dual signal amplitudes to one another. This may reveal the relative contributions of the thoracic and abdominal ribcage in a two-component model. Although we attempted to measure this variable in the current study, signal noise presented methodological concerns. Future investigations might consider other noise reduction techniques or sensor application strategies to mitigate these problems and evaluate this potentially valuable element of BP.

Finally, although we report highly accurate FR detection, we did not integrate this into LRC analysis in our study. Such analysis would require combination with step detection, which was not the purpose of this investigation. HX does include an accelerometer and provides raw data and factory processed step timestamps. Subsequent studies could examine LRC versus a reference system (e.g., spirometer and instrumented treadmill) to expand this knowledge.

### 4.3. Limitations and Practical Applications

These results demonstrate that a field-ready wearable such as HX can provide valuable FR and BP detection versus criterion measures. Unfortunately, using a spirometer as the reference device confined the study environment to a sports science laboratory, and treadmill running may elicit different BP responses than field running [5,32]. Furthermore, spirometry masks change runners’ natural BP [33]. Hence, we recommend caution when considering the ecological validity of these results, and simultaneously highlight the capacity of HX and similar stretch respiration sensors for avoiding these detrimental measurement factors.

Our work reinforces the importance of proper sensor fitting and adjustment during studies and field use. Previous reports highlighted the importance of adherence to manufacturer sizing (instead of self-selection by the wearer) and use of the provided circumferential elastic bands, among other recommendations for optimal HR and other data [9,27].

## 5. Conclusions

This study sought to determine the FR and BP detection accuracy of the HX and custom algorithm versus a reference spirometer during running. Our results demonstrate enhanced capabilities for the HX system to provide accurate and valuable BP data when combined with a custom FR detection algorithm. More method comparison studies are needed to compare other components of BP between wearable sensors and reference systems, and to translate these findings into the field.

## Figures and Tables

**Table 1:** Participant characteristics.

Variable	All *n* = 12	Females *n* = 6	Males *n* = 6
Age (y)	31.5 ± 4.5	32.6 ± 5.9	30.5 ± 2.8
Height (m)	170.9 ± 10.7	165.3 ± 8.6	176.5 ± 10.2
Mass (kg)	63.4 ± 12.7	56.6 ± 10.0	70.3 ± 11.8
Sum of torso skinfolds (mm)	29.9 ± 6.9	30.8 ± 8.1	29.0 ± 6.1
Ratio chest:abdomen	1.1 ± 0.1	1.1 ± 0.1	1.1 ± 0.1

**Table 2:** Group sample characteristics.

Variable	Source	Observations	Minimum	Maximum	Mean ± SD
tI (s)	CM	3907	0.12	2.63	0.76 ± 0.25
	HX	3907	0.28	2.81	0.81 ± 0.26
tE (s)	CM	3907	0.17	3.21	0.86 ± 0.29
	HX	3907	0.26	3.02	0.81 ± 0.27
tB (s)	CM	3906	0.75	5.36	1.61 ± 0.51
	HX	3906	0.73	5.26	1.62 ± 0.51
BR (bpm)	CM	3906	11.2	73.0	40.5 ± 11.2
	HX	3906	11.4	71.7	40.3 ± 11.2
ds (%)	CM	3906	30.8	69.6	47.1 ± 5.0
	HX	3906	28.3	73.2	49.9 ± 3.0
BRV (%)	CM	3838	0.0	218.4	80.0 ± 32.1
	HX	3838	0.0	208.8	77.6 ± 32.6

**Table 3:** FR detection accuracy.

p	TP	FP	FN	Precision	Recall	Lag (s)
P1	742	0	3	1.000	0.996	0.016 (−0.051,0.083)
P2	667	0	0	1.000	1.000	0.062 (0.013,0.112)
P3	496	1	0	0.998	1.000	−0.011 (−0.046,0.024)
P4	887	5	1	0.994	0.999	0.037 (−0.056,0.131)
P5	674	0	0	1.000	1.000	−0.064 (−0.109,−0.020)
P6	600	0	0	1.000	1.000	0.013 (−0.029,0.054)
P7	469	0	0	1.000	1.000	0.093 (0.027,0.160)
P8	479	0	2	1.000	0.996	−0.107 (−0.196,−0.019)
P9	876	6	0	0.993	1.000	0.090 (0.042,0.138)
P10	527	0	4	1.000	0.992	0.002 (−0.063,0.068)
P11	598	3	1	0.995	0.998	0.007 (−0.049,0.063)
P12	801	0	0	1.000	1.000	−0.101(−0.152,−0.050)
Pooled	7816	15	11	0.998	0.998	0.018 (−0.067,0.104)

Notes. p: participant; TP: true positive; FP: false positive; FN: false negative.

**Table 4:** Method comparison results.

Variable	Bias	95% LOA	Intercept	Slope	MRPE (%)	MAPE (%)	r
tI (s)	0.051 (0.049,0.054)	(−0.112,0.215)	−0.026 (−0.038,−0.015) **	1.11 (1.09,1.12) **	6.45 (−2.85,15.76)	8.65 (1.35,15.95)	0.95 **
tE (s)	−0.045 (−0.048,−0.041)	(−0.248,0.158)	0.066 (0.056,0.076) **	0.87 (0.86,0.88) **	−4.99 (−13.68,3.71)	7.76 (1.42,14.1)	0.94 **
tB (s)	0.007 (0.003,0.010)	(−0.184,0.197)	0.002 (−0.009,0.013)	1.00 (0.99,1.01)	0.27 (−3.97,4.52)	2.74 (0.00, 5.99)	0.98 **
ds (%)	2.79 (2.64,2.94)	(−5.72,11.31)	31.75 (28.94,34.55) **	0.39 (0.33,0.45) **	6.13 (−2.78,15.03)	8.10 (0.94,15.26)	0.50 **
BR (bpm)	−0.19 (−0.27,−0.10)	(−4.89,4.52)	0.148 (−0.092,0.387) **	0.99 (0.98,1.00) *	−0.27 (−4.52,3.97)	2.74 (0.00,5.99)	0.98 **
BRV (%)	−0.23 (−0.34,−0.11)	(−6.72,6.26)	0.24 (0.16,0.32) **	0.95 (0.92,0.97) **	0.77 (−49.85,51.38)	36.24 (0.91,71.57)	0.92 **

Notes. LOA: limits of agreement; MRPE: mean relative percent error; MAPE: mean absolute percent error; r: Pearson’s correlation coefficient. Significance for intercept and slope denote value different than 0 or 1, respectively. * *p* < 0.05, ** *p* < 0.001.

## Data Availability

The datasets generated for this study are available on request to the corresponding author.

## Abbreviations

BP	Breathing pattern
BR	Breathing rate, respiratory frequency (breaths per minute, bpm)
BRV	Breathing rate variability (%)
CM	Cosmed Quark spirometer
ds	Duty cycle, breath ratio (inhale time/breath cycle time) (%)
FN	False negative
FP	False positive
FR	Flow reversal
HX	Hexoskin smart shirt
LOA	Bland–Altman 95% limits of agreement
LRC	Locomotor-respiratory coupling
tB	Breath cycle time (from inspiration to next inspiration) (s)
tE	Exhale time (s)
tI	Inhale time (s)
TP	True positive
